# Hahnemann on Large Doses of Homœopathic Remedies

**Published:** 1884-10

**Authors:** 


### HAHNEMANN ON LARGE DOSES OF HOMŒOPATHIC REMEDIES.

" Large doses of remedies which are homœopathically indicated are much more certainly injurious than when they are given without holding any relation of similarity (homœopathic relation) to the case of disease, or when they stand in a relation of contrariety (antipathic), that is, are given entirely at random (allopathically). In the homœopathic use of remedies, in cases where the totality of the symptoms of the patient is paralleled in great similarity by the action of the drug, it is a real crime not to give very small, the smallest possible, doses; for in these cases massive doses, such as are ordered of drugs in the ordinary quack practice, are veritable poisons and murderous potions. Convinced by thousand-fold experience, I make this declaration, and mean it to apply to every homœopathic administration of drugs in general and *universally*, especially when the disease is acute * * * Let no one come then and say * * * a remedy has been given * * * in an appropriate case, yet in the strongest doses and not too seldom either, but every two or three hours, and *yet* the patient is dead. Nay, I reply, from full conviction, *for that very reason* he is dead, and thou hast killed him. Hadst thou given him a single dose of the smallest part of a drop of the twenty-fifth or thirtieth dilution (in rare cases a second dose, repeated on the third or fourth day), then had the patient been saved, *certainly* and with much less trouble."—*From Note to Prov. of Hyos., Reine Arzneimitretellehre*, 4, 46. 1825.

